# Modus Operandi and Effects of Quinine

**Published:** 1847-03

**Authors:** M. H. Fee

**Affiliations:** Leatherwood, Lawrence county, Indiana; Heltonsville


ART. IV.—*Modus Operandi and Effects of Quinine.* By DR. M. H FEE, Leatherwood, Lawrence county, Indiana.

Think not, reader, when you see this caption, that I design to explain the vis predilectio of the various organs of the animal economy, by which, as if by chemical affinity, one article of the materia medica is irresistibly drawn to a particular organ, while another article, by this organ, is repelled and forced to seek its affinitive somewhere else. If you come to this conclusion, I am sorry to inform you that you will be sadly disappointed. It does not belong to my province to say why the specific action of terebinthina is spent upon the kidneys rather than upon the heart; or that of secale cornutum upon the gravid uterus rather than upon the bladder; or that of the absorbed virus of cantharides, from a blistered surface, upon the kidneys and neck of the urinary vessel rather than upon the lungs; or why that of aloes should be spent upon the lower bowels rather than upon the upper. These are mysteries beyond my depth, and I leave them for others to speculate curiously upon, with the simple remark, that "they are so, because God made them so;" and here, for the present, our knowledge must rest. My business is to show the effects of quinine on the various parts of the system, and how those effects are produced; not why it affects particular parts rather than others. In the following pages, I design to establish, as clearly as it is possible to do, short of ocular demonstration—1st, that quinine in small doses is tonic, and how this tonic effect is produced; 2d, that it is antiperiodic, and how this is effected; 3d, that in large doses it is directly sedative, and how these effects are produced; 4th, that it possesses anti-congestive properties, and how it removes congestion; and, 5th, that it possesses no narcotic properties.

Let me simply state that the following thoughts are the fruits of some years' patient investigation and close observation. I give them to the profession for what they are worth. I flatter myself that in the present state of medical knowledge with regard to this article, my observations will not be cast into the common pile of professional rubbish before they have had an impartial hearing. Now to the subject. I proposed—

1st. To establish that quinine is tonic in its effects. That it possesses tonic properties in an eminent degree, when administered in the proper doses (from one to two grains three or four times per day), is amply proved, firstly, by the return of nervous and consequently muscular strength, lost by previous indisposition; secondly, by the replacement of steadiness of brain and nerve, instead of the bedizened state of the former and tremulous state of the latter; thirdly, by the rapid and vigorous reestablishment of the digestive functions and peristaltic action of the bowels, by which perfect digestion and regular alvine dejections are restored; and, fourthly, by the increased power of the heart, by which the flagging and contracted pulse is converted into the full and soft. These various effects are produced by the stamina which is given to the nervous system by quinine, by which is prevented the return of those pathological conditions of the system which constitute disease. I mean particularly capillary congestion for want of nervous power. Quinine lends to the weakened nervous system the power to compel the various organs to perform their functions—to break down all obstruction—to overleap every obstacle. The thus renovated nervous system influences the heart to throw a greater amount of blood to the brain in a given time, by which its bedizened state is fully dispelled. It now accumulates strength in an exact ratio with the increase of its proper nutriment. This increase of energy is then transmitted to the lungs, the consequence of which is increased powers of respiration, by which the oxidation of the blood is more perfect; the consequence of which is a still more wholesome aliment to the brain, enabling it to engender the nervous fluid in fourfold bounty, which it sends with electric speed to all parts of the system, revivifying and reanimating the broken down energies of the system. Thus they go on—brain acting on organs, and the latter on the former, till the health of the whole system is perfectly reestablished.

2d. I proposed to establish that it is antiperiodic. I need say but little on this point to establish what all concede. None, at this enlightened period of its history, are in doubt of its antiperiodic properties. But how this effect is produced there is perhaps not so much unity of opinion. Therefore, to some my thoughts, though perhaps not interesting in point of value, may be as a source of amusement. Let me, for the sake of being fully understood, give my views of the cause of periodicity of some forms of disease. I consider every form of periodic disease, as being *purely* nervous in its origin, and, frequently purely so, throughout its whole course.

The exciting cause of periodic disease, whatever it may be, makes its impression upon the nervous system. Frst, by prostrating nervous energy, which is usually called the first stage, or stage of oppression. This is the only stage in which the exciting cause may, properly, be said to act; the subsequent stages being the results of action of the nervous system, independent of the exciting cause. Second, by producing nervous *functional* lesion, the consequence of which is perverted, or suspended secretions. And, third, sometimes by producing nervous *structural* lesion, the consequence of which is, almost immediate death. Now if the first obtains, an intermittent will be the consequence; if the second, a remittent; if the third, the most rapid and fatal form of congestive disease; such as Johnson tells us of, in his "Diseases of tropical climates." Then, that a periodic disease may be produced, it is necessary that the exciting cause be weak, or that the invaded system be strong. As a matter of course if the exciting cause meet with strong resistance, it cannot make so great an impression, and vice versa. Then periodicity is brought about by alternate preponderance of the exciting cause and the powers of the system. When the exciting cause begins to invade the system, it brings the powers of the system gradually under its control until it at last, almost, reigns predominant. The tyrant, however, holds his advantage but for a short time. The outposts are but driven in to cooperate with the main force, a sharp conflict ensues, and the enemy is repulsed. But he sees that he has made an impression. If he has considerable force and finds that he has made considerable impression, he will renew the fight in twenty-four hours. But if his forces are weak and the impression on his enemy in the former struggle but slight, he will postpone his next attack for thirty-six, forty-eight, or seventy-two hours. Thus the belligerents go on fighting periodically, until, from sheer necessity, the one or the other is forced to capitulate. Sometimes, however, the system, from force of habit, will fight its battles over and over again, when there is really no enemy in its territory; or, may be, it is a very prudent, warlike demonstration it makes to hold a conquered but subtle foe in subjection. One thing is certain: the system on these occasions is very careful not to uselessly expend any great amount of ammunition.

Pardon this digression; it was made for the purpose of making plain my views. Now to explain how quinine puts an end to hostilities: It always acts as an ally with the system, either as an ally of force or that of stratagem. As an ally of force, it assists the system by hourly joining detached forces to those of the system, commencing several hours before the expected attack. It urges the dispirited troops to strengthen their weakened fortifications, to rally their sinking spirits fo a gallant defence, to throw away encumbrance. In a short time, the united forces have made considerable improvement in their defensive structure; they now shout defiance to the distant enemy. Presently the bugle of the advance guard of the enemy gives the signal of attack; their topographical engineers reconnoitre the allied forces' entrenchments; they see the bustle, animation and determination of the allied army; they report their observations to their commander, who orders a few shells to be thrown, abandons the conflict, calls off his forces, and proclaims the independence of his enemy.

Now to present quinine in the character of an ally of stratagem: He persuades his ally to assume the appearance of a dead army. Here they are, in pursuance of his instructions, all apparent feebleness; they show but little signs of life, less of resistance. General Quinine now retires behind the screen to observe the success of his stratagem. The enemy shortly heaves in sight; they form their forces in battle array; the bugle sounds the charge; they rush to the breastworks, and peer over them with malice in their eyes—when, lo! they behold a prostrate and apparently lifeless foe. They are too magnanimous, too tenacious of military etiquette, to dishonor their weapons by striking a fallen foe. They return and report their foe 'defunct.'

Need I add one word of explanation of this figure? It needs it not. I shall simply say that quinine, given in one gr. doses for seven or eight hours prior to the expected paroxysm, prevents it, by giving the system power to prevent the return of the various pathological conditions of the system which favor a repetition of the paroxysm. In large doses (twenty grains) immediately before the paroxysm, it prevents it by its quieting influence on the nervous system, which, according to my theory, renders the system noninvasive by periodic disease. This will be more fully explained under the next head. Morphine and the various preparations of opium, and also others of the narcotics, have the power of preventing the recurrence of periodic disease, though not to the same extent as quinine, because they do not possess equal anticongestive powers. To close this head, I shall draw the points of difference between the action of small and large doses of quinine, and how they, acting differently, produce the same effects. But few words are needed. Small doses give tone to the nervous system, to prevent the return of nervous irritation, by actual gain of energy. Large doses have but little tonic effect; consequently we cannot attribute its antiperiodic effects to actual strength imparted; therefore we must attribute its antiperiodic powers to its quieting influence, by which all nervous irritation is kept down till the paroxysmal time passes.

3d. I proposed to show that quinine is directly sedative. The evidence I bring to bear on this point is, first, that when administered in doses of fifteen to twenty grains two hours apart for two or three doses, there is great diminution in the fullness and frequency of the pulse. I have often sat by a patient and felt his pulse sink from twenty to thirty beats in the minute, in the space of two hours, under the administration of this article; whereas, had I not given some sedative, the pulse would have remained stationary, or perhaps have risen. Second: When carried too far, that is, when the system is supersaturated with quinine, great prostration occurs, with small, rapid and weak pulse, scarcely audible motion of the heart, and anxious, hasty and imperfect respiration. These are positive proofs of its sedative powers. Another weighty proof in corroboration of this property is that one or two twenty-drop doses of laudanum will effectually relieve the prostration. Morphine, given in conjunction with quinine, greatly modifies its sedative properties, and has the effect of gaining the desired end with a less outlay of quinine. These sedative effects are produced through the power quinine possesses of lowering excitability by taking away or destroying the impressibility, to a great degree, of the nervous system. This view of the subject is favored by the great length of time required by a fully quininized patient to regain his usual impressibility and standard elevation of pulse. In its effects it appears to be nearly akin to tartar and digitalis.

4th. I proposed to establish that quinine is anticongestive. I establish this fact from the following observations, viz.: First, that a few full doses of this article will immediately effect great and permanent reduction of an engorged spleen. Second, that a dull, heavy, aching pain of the liver, which is certain evidence of a congested state of this viscus, is removed by a few full doses of quinine. Third, that the pain always found in bilious fever, in the region of the kidneys, is quickly removed through the agency of this article. Fourth, that the scanty secretion of the liver is soon followed by copious bilious stools shortly after the administration of a laxative, when quinine has been freely used. Fifth, that the secretory action of the kidneys is reestablished, whereby a free and copious discharge of urine takes the place of the painful and scanty discharge of this secretion. Now, these anticongestive effects cannot be produced with small doses of quinine, unless congestion be the consequence of actual weakness of nervous energy. Let me here be understood. There are two conditions of the system in which congestion may take place: Firstly, where there is a weakened state of the system from previous disease, an actual lack of nervous energy to keep up the capillary circulation throughout the various viscera; and, secondly, where the nervous energy is normal, but the nerves are too irritable. The effects will be precisely the same, but the treatment is different. The former condition will require tonics and stimulants, while the latter will require something to reduce excitability. Quinine in small doses will be proper in the first condition of the system; but in the latter, mammoth doses must be given, combined with morphine. Quinine in small doses, in a weakened condition of the nervous system, gives power to the nervous system and through it to the circulatory vessels, to prevent stagnation in the capillaries. Where congestion arises from irritability of the nervous system, quinine in large doses acts not by lending power to the nerves to force the blood through the capillaries; but by quieting nervous irritability, it allows the spasm of the coats of the vessels to cease, and the circulation to be carried on normally by the ordinary power of the heart. I think I am now understood.

5th, and last. I proposed to show that it possesses no narcotic powers. Some late writers urge the following symptoms as evidence of its narcotic action. First, that when given in considerable doses, it produces ringing in the ears; partial deafness and blindness. Second, that it produces flushed face with fullness of the vessels of the head. I shall endeavor to account, in a more rational manner for these symptoms, than to attribute them to narcotism. The ringing in the ears, with partial deafness and blindness, are caused by the reduction of power the quinine effects in the nervous energy of the heart, by which less blood is thrown to the brain in a given time, consequently the brain receives less of its proper nutriment, which produces an anemic state of the brain, such as would occur in an excessive loss of blood. This is the work discernible, by being contrasted with the hyper excitement of the brain, just before, from the influence of fever. There is one fact which I can bring to prove the correctness of my position. When the brain is in this state, if the erect position be assumed, all the symptoms will be increased, but if the head be let down even with the shoulders, the symptoms will be ameliorated. The flushed face may be accounted for, in the same way that the injected eye, and pseudo-inflammation of the brain are accounted for, in excessive losses of blood. Though in complete quininism I have never observed this state of the face. Where it has been noticed, I presume the dose has not been sufficiently large to give play to its sedative properties. In full quininism, I have invariably found an exsanguine countenance, cold hands and feet, wlth overloaded heart, lungs, and large arterial trunks. In short, a want of power in the heart to carry on the circulation.

Heltonsville, Ind., Dec. 14th, 1846.

